# Glucose Sensor Using Sol–Gel Coating Layer Deposited on PMMA Optical Fiber: An Enzyme Activity Measurement System

**DOI:** 10.3390/gels9080608

**Published:** 2023-07-27

**Authors:** Jorge-A. Ortega-Contreras, Edgar Alvarado-Méndez, Guillermo Almanza-Rodríguez, María del Carmen Hernández, Luis Celaya-García

**Affiliations:** 1 Department of Electrical Engineering, DICIS, Universidad de Guanajuato, Salamanca 36787, Mexico; ja.ortegacontreras@ugto.mx; 2 Department of Electronics Engineering, DICIS, Universidad de Guanajuato, Salamanca 36787, Mexico; 3 Department of Biochemical Engineering, Tecnológico Nacional de México, Celaya 38010, Mexico; galmarod@gmail.com (G.A.-R.); carmen.hernandez@itcelaya.edu.mx (M.d.C.H.); 4 Department of Mechanical Engineering, DICIS, Universidad de Guanajuato, Salamanca 36787, Mexico; ld.celayagarcia@ugto.mx

**Keywords:** sol–gel, glucose, biosensor

## Abstract

In the present work, a biocatalytic glucose optical sensor produced by immobilizing glucose oxidase (GOD) as a recognition molecule over a PMMA (polymethylmethacrylate) optical fiber is introduced. An enzymatic encapsulation process was carried out using the sol–gel method, depositing a TEOS-based coating by immersion at the end of an optical fiber; the biosensor was characterized using different glucose levels. Finally, the best way to encapsulate the enzyme and prevent it from degrading is to perform the process at room temperature, and later implement the deposition of the coating on the fiber. The drying process was optimal below 8 °C.

## 1. Introduction

According to the International Union of Pure and Applied Chemistry (IUPAC), a biosensor is a device that employs specific biochemical reactions through enzymes, antibodies, tissues, organelles, or whole cells (recognition element) to detect chemical compounds (analyte), typically through electrical, thermal, or optical means [1]. This type of sensor is based on the immobilization of proteins or enzymes that can be adhered to a solid insoluble matrix, to protect the enzyme from self-degradation and microbial agents. Immobilization allows for transducers to be prepared such as electrodes, piezoelectric, field-effect transistors, and optical, among others [2]. Optical sensors have advantages over other methods such as immunity to electromagnetic interference, compact size, cheapness, remote sensing, multiplexing, etc.; therefore, immobilization for optical sensing is of great interest. Enzymes can be immobilized by two methods: reversible and irreversible [3]. Irreversible immobilizations can be by covalent bonding, multi-point covalent attachment, and entrapment. The reversible method is based on adsorption. Sol–gel is a technique of immobilization of enzymes on some dye, which is used in the manufacture of transducers. Inert films can be made more resistant than polymer films to abrasive environments. Several properties of sol–gel glasses make them particularly appealing as suitable environments for enzymatic catalysis: (a) the capacity to trap large amounts of additives, (b) the thermal and chemical stability of the matrix, (c) the simplicity of preparation without covalent modification, (d) easy manipulation and technological production in any desired geometry, including thin films. The application of the sol–gel process [4] to entrap organic macro-molecules in inorganic glasses is now well-documented and extensively demonstrated. A brief overview of the state of the art is presented in [5], which compiles examples of some common chemical sensors and introduces a ruthenium fluorescence sensor entrapped in sol–gel. This technique has also been employed for the creation of long-term flexible sensors using electrochemical deposition. In [6], the authors explain creating an iridium oxide microelectrode for the indirect bio-electrochemical detection of dopamine. Their sensor exhibits less pH dependence compared to other electrodes fabricated through electrochemical deposition.

Over the past few decades, the development of inorganic sol–gel matrices with encapsulated enzymes has emerged as an efficient method for the production of easily recyclable biosensors. Silica gels ($SiO_{2}$) are the most commonly used due to their ability to form a porous gel network around each enzymatic macro-molecule present, encapsulating them within the pores and allowing the passage of substrates through the pores [7]. In this concept, the enzyme has freedom of movement within the pore. Therefore, the enzyme molecule does not need to be bound by adsorption or other ionic or covalent bonds to the inner walls of the gel, although such interactions may arise during the process and interfere with the enzyme efficiency. The key steps for proper enzyme encapsulation involve precise control of the pH, temperature, solution buffer, and gel condensation time for efficient pore sizes. In [8], the creation of a direct glucose sensor by encapsulating GOD in $SiO_{2}$/TEOS silica gel, coupled with an electrochemical oxygen detector, was reported. It is considered a direct detector as it can measure the concentration of molecular oxygen ($O_{2}$) present in the medium, which decreases over the course of the enzymatic reaction time. [9] documents various biosensors that have been reported for the past three decades, including $H_{2}O_{2}$ and $O_{2}$ electrodetectors, redox detectors, fermentation detectors, and oxidizing and reducing analyte detectors using different enzymes, including GOD, in various types of encapsulation. In this work, a method for producing a xerogel-sensitive layer through the encapsulation of GOD via $SiO_{2}$/TEOS sol–gel on a PMMA optical fiber is presented. Additionally, the activity of GOD in the xerogel was measured by coupling the biosensor with redox detection using horseradish peroxidase (HRP) and o-Dianisidine as a redox dye, which is measurable at an absorbance of 532 nm.

## 2. Results and Discussion

### 2.1. Coating

**Figure 1:**
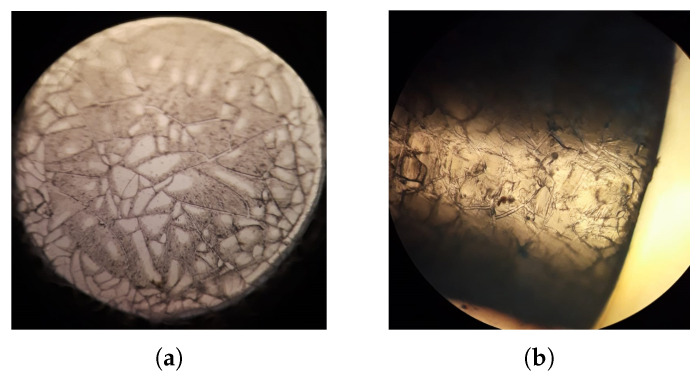
Bioptrode observed under 50x objective. (**a**) Front view (**b**) Side view.

**Figure 2:**
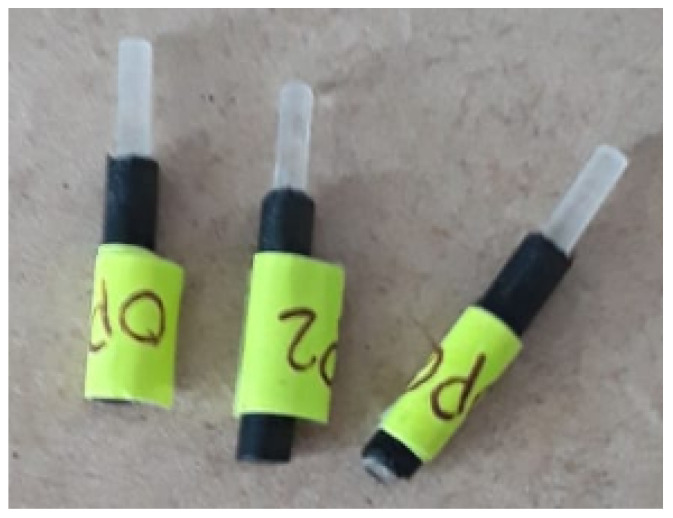
Bioptrodes.

After the completion of the test, the scratch track was analyzed by using a microscope to look for damage such as delamination, bulking, and spallation. After observing several experiments to test the sol–gel adhesion without enzyme encapsulation, the best result (R1) was obtained with the following quantities TEOS: 5 mL, CH${}_{3}$CH${}_{2}$OH: 5 mL, HCl (0.1 M): 0.8 mL stirred for 20 min and dried for 16 h. The pH and the drying conditions influence directly on the grain size of the coating [10,11]. In the sol–gel process, the particles in the sol can lead to fibers, monoliths, powder, and coating films. In all these cases, a process to control the final product in the sol–gel transition can be chosen. Lower values of pH speed up the hydrolysis process (acid hydrolysis), but the polycondensation (dehydration and dealcoholization) reaction will slow down. Both processes occur at the same time, the ROH groups produced in the hydrolysis are used in the polycondensation, and some amount of alcohol and water will be evaporated. Using a higher pH the gelation time is dramatically reduced but the final product after the transition will be a powder. Once the gelation has occurred, the final step is the drying process. This can be under supercritical conditions to produce aerogels, or low-temperature evaporation of the solvent with the addition of a drying control chemical additive, in which case a xerogel can be obtained. The effects of removing the water/solvent in the gel are related to the pore size. Using the colloidal method, a mesoporous membrane is produced. The insertion of active enzymes defines the physical limit of the average grain size. Figure 1 shows the doped GOD coating over a plastic optic fiber (POF). The coating does not exhibit any damage. In Figure 2, some plastic optic fibers coated with sol–gel are presented.

### 2.2. Adhesion Test

An analysis of experiments was conducted to find the optimal adhesion of the coating to glass slides. Here, the amount of water in the solution is changed, the excess water acts to inhibit the condensation reaction, and minimize the pH of the solution. The pH is a determinant factor in controlling the gelling time. In Table 1, the volume composition of the assays used to test the adhesion can be observed.

To select the solution with the best adherence, slides were dip coated at different times during the stirring process. Starting with variations of twenty minutes until two hours of mixing, then every two hours, until completing eight hours of mixing, and finally eight more hours. Once the coating was dry, a scratch test [12] was conducted. Different errors appeared because of the properties of the substrate and coating; however, we were looking for spallation and bulking errors. Using an ’H’ pencil [13] a grid of 5 mm × 5 mm was drawn over the surface at nearly constant normal force and velocities.

### 2.3. Colorimetry Assay

**Figure 3:**
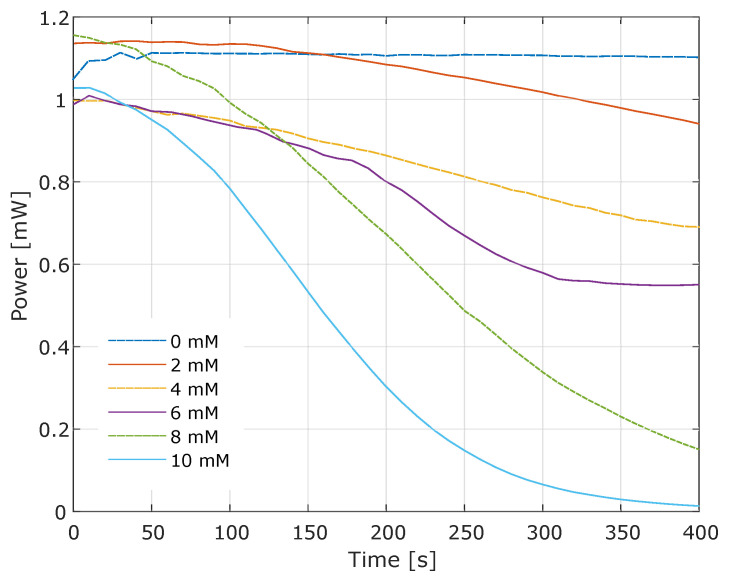
Effect of the GOD/HRP staining reaction with o-Dianisidine on power.

The enzymatic activity of GOD can be measured by colorimetry [14] based on the GOD/HRP enzymatic reaction with o-Dianisidine as a color indicator. Horseradish peroxidase (HRP) utilizes the peroxide produced during the glucose oxidation catalyzed by GOD. The o-Dianisidine molecule is oxidized in the presence of HRP, resulting in an orange-amber coloration [15]. In this assay, six solutions with different glucose concentrations ranging from 0 to 10 mM were used. As the concentration increased for each solution, a greater decrease in power over time was observed. The coloration in the solution, caused by the oxidation of o-Dianisidine, is proportional to the power drop. Higher glucose concentrations lead to increased turbidity in the solution. The point of maximum turbidity for each concentration indicates the end of the reaction and the linearity of the final power (Figure 3). Time is measured in seconds (s) and power in milliwatts (mW).

**Figure 4:**
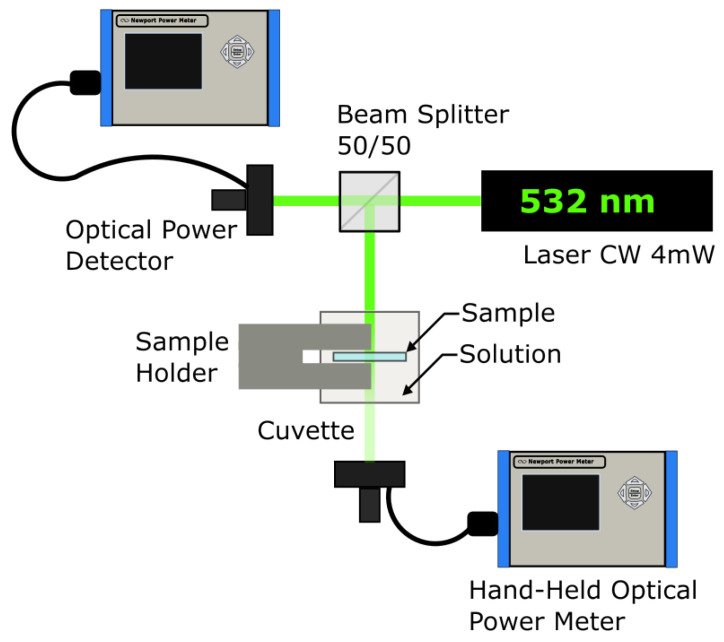
Optical arrangement for colorimetry assay.

The power drop is faster at higher glucose concentrations due to the substrate concentration gradient approaching the GOD’s Michaelis constant (Km) at each step. In the GOD/HRP reaction system, the GOD enzyme exhibits the slowest reaction rate. The color change in the samples was measured using the setup depicted in Figure 4, capturing the power measurement every 10 s for all samples. The measurement instruments used were the Newport model 1918-C hand-held optical power meter and the 918D-SL-OD1R silicon photodetector. From the power data, the absorbance curves for each concentration were calculated using Equation (1).

(1) $$\begin{array}{ccc} A & = & -log_{10}\frac{I}{I_{0}} \end{array}$$

**Figure 5:**
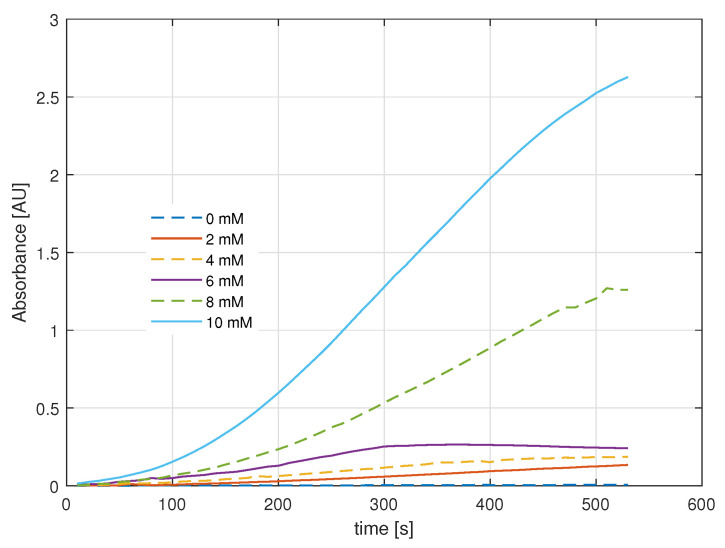
Absorbance in the colorimetry assay.

Unlike the power drop curves, the absorbance curves indicate the change and intensity of the color of the samples with an upward trend proportional to the amount of light absorbed by the oxidized o-Dianisidine (Figure 5). Based on the absorbance data, the reaction rate for each concentration can be estimated as Equation (2).

(2) $$\begin{array}{ccc} v & = & A/t_{lin} \end{array}$$

**Figure 6:**
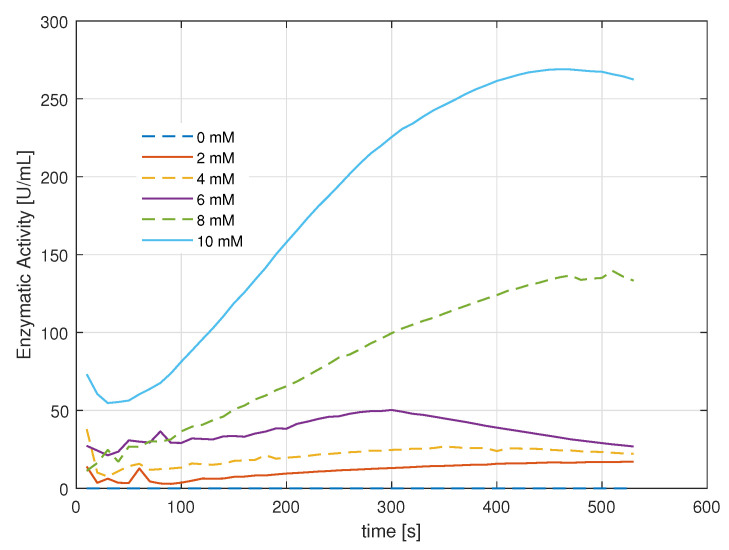
HRP enzymatic activity.

Finally, the enzymatic activity was calculated in terms of enzyme units per milliliter of the sample solution, with the 0 mM solution used as the blank. The calculation is performed according to Equation (3), where $V_{assay}$ is the volume in milliliters of the sample, df is the dilution factor, κ is the millimolar extinction coefficient of oxidized o-Dianisidine at 532 nm, and $V_{enzyme}$ is the volume in milliliters of the enzyme used. The enzymatic activity is presented in Figure 6.

(3) $$\begin{array}{ccc} \mathrm{Units}/\mathrm{mL}\;\;\mathrm{enzyme} & = & \frac{\left(\Delta A_{532\mathrm{nm}}/\min\;\mathrm{Test}-\Delta A_{532\mathrm{nm}}/\min\;\mathrm{Blank}\right)V_{\mathrm{assay}}\;df}{\kappa_{\mathrm{at}\;532\mathrm{nm}}V_{enzyme}} \end{array}$$

The units of active enzyme per milliliter of each concentration show the sensitivity of the sol–gel-fixed GOD/HRP to low substrate concentrations. One unit will oxidize 1.0 μmole of β-D-glucose to D-gluconolactone and $H_{2}O_{2}$ per minute at pH 5.1.

**Figure 7:**
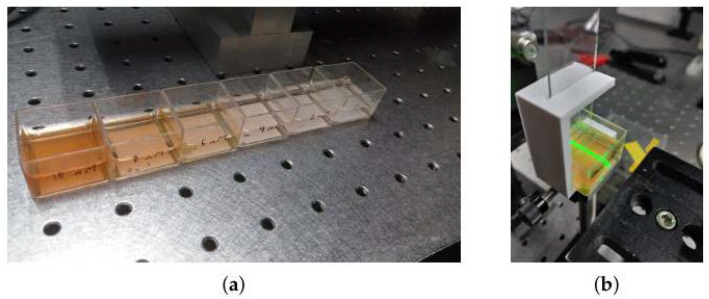
Colorimetry assay tests. (**a**) Saturation color scale from left to right 10 mM to 0 mM. (**b**) Power measurement at the highest concentration.

Figure 7a shows the color scale in descending order for the concentrations used; Figure 7b shows the color saturation point for the 10 mM sample with the incident 532 nm green light beam.

### 2.4. Biosensor Analysis

**Figure 8:**
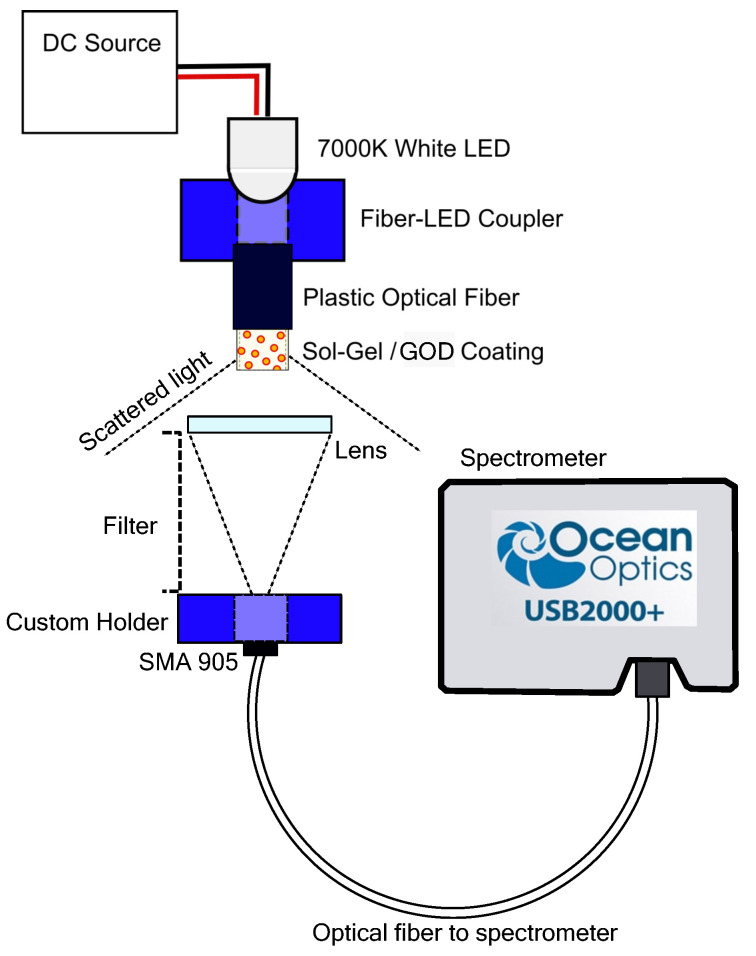
Schematic of experimental setup in transmission mode to test the bioptrode.

The characterization of the biosensor was performed by preparing different glucose solutions in distilled water at various concentrations. The biosensor can be used in different optical configurations [16]; the more common application for an optical fiber is in transmission mode. The sensitive region of the optrode will be exposed to the ambient light. Because of the change in the medium refractive index, the light will be scattered. Nevertheless, it is obligatory to use a double convergent lens, at adequate distance [17], to couple the radiant power into the multi-mode fiber-optic connector (SMA905). The spectrometer will further analyze the coupled power. The optical setup employed to measure the data is shown in Figure 8. A custom-made plastic coupler was used to guide the light inside the optical fiber. In [18], a system to determine the absorption spectra in scattering media using a light emitting diode (LED) was proposed. The light source used in the setup presented in this article is a broad-spectrum LED. The power source of the LED was a constant-current source with temperature compensation to minimize systematic errors.

The concentration range spanned from 100 mg/dL to 200 mg/dL, with intervals of 10 mg/dL. Hence, eleven different concentrations were prepared. All the test equipment can introduce noise to the measurements, in optical components, a common source of error is the thermal noise and the shot noise [19], and they can appear in the CCD of the spectrometer or the photodetector. The transmission spectrum of the bioptrode was calculated from 100 samples measured with the spectrometer using a sampling time of 100 ms.

### 2.5. Spectrophotometry

**Figure 9:**
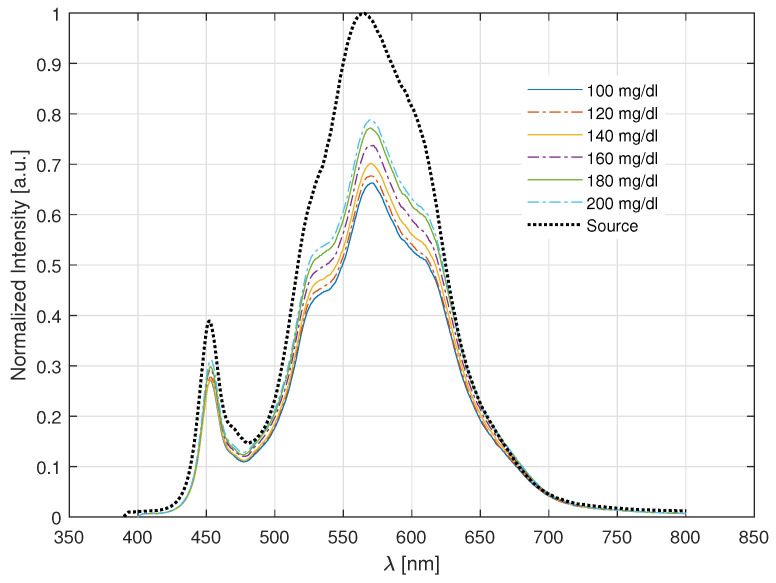
Normalized intensity at the output of the POF.

In this section, the performance of the bioptrode with a light source in the visible range will be presented. The characterization of a glucose biosensor was developed by studying the relationship between the glucose concentration and the optical signal that passes through a plastic optical fiber. Fiber-optic sensors work by the measurement of optical properties such as absorption, transmittance, fluorescence, evanescent wave, etc. The light travels through the POF and finally arrives at the sensitive region, the doped sol–gel coating. The collision between matter and light is complex, because it is inelastic, changing the rotational and vibrational energies of the molecules. There is no satisfactory mathematical model that can fully account for all the phototransduction mechanisms. Here, we attribute the sensor’s behavior to two principal components: the change in refractive index *n* (RI) due to the oxidation produced in the POF tip; and to a possible weak fluorescence of the oxidized enzyme [20,21]. The dominant phototransduction phenomenon is the change in the RI, it mainly changes the scattering properties of the media [22]. The intensity of the reflected beams and the value of *n* are directly proportional. Different glucose concentrations will be deposited on the tip of the sensor using a micropipette. The chemical reaction with the glucose changes the transmittance power, as seen in Figure 9. At the biosensor–air interface, the highest power transfer is observed. This is due to the difference in the refractive index of the materials [23]; PMMA has n=1.49 and air n=1.0003, then, a tiny part of the light will be dispersed. All the glucose concentrations have an RI close to that of water n≈1.333. The RI difference PMMA–water is lower, thus, the transmitted power will be reduced due to the increase in power of the reflected beam. This can be noticed in Figure 9. At a concentration of 100 mg/mL, the power is lower than the source. The GOD immobilized in the tip of the biosensor oxidizes the glucose, increasing the imaginary part κ of the RI and lowering the reflections. The oxidation increases the transmission power.

**Figure 10:**
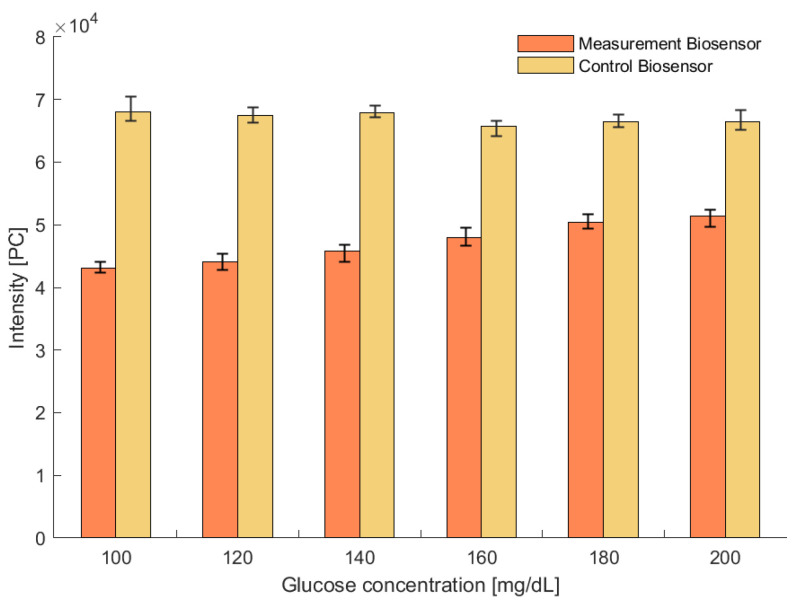
Statistical analysis of the sensitivity of the biosensors’ intensity to exposure to different analyte concentration levels.

The linearization of the sensor was made at the wavelength with the largest difference with respect to the source; this happens at λ=568 (nm). The photon count (PC) measured by the spectrometer is shown in Figure 10. The data in this figure correspond to four biosensors from the same batch; after each set of measurements the biosensor was cleaned in distilled water. The test was conducted by raising the analyte concentrations.

**Figure 11:**
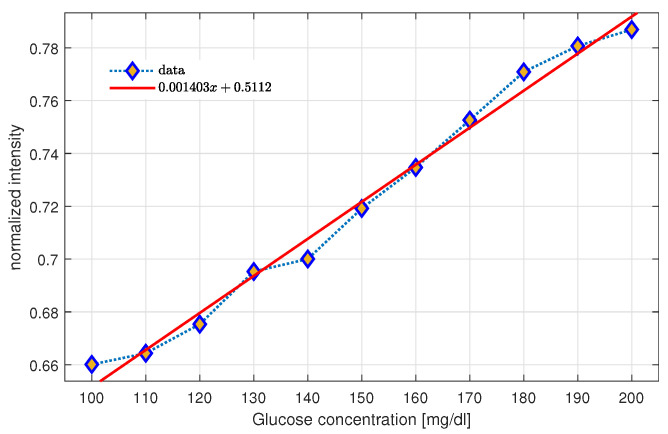
Linear regression.

For an average blank level of ${\mathrm{mean}}_{blank}=6.4464\times 10^{4}$ with a standard deviation of $\sigma_{blank}=7.118\times 10^{2}$, the analytic blank level of the biosensor is $\mathrm{LoB}=6.2328\times 10^{4}\;\left(PC\right)$. The lowest analyte concentration distinguishable from the LoB is computed from $\sigma_{init\;\;sample}=7.6909\times 10^{2}$, resulting in $\mathrm{LoD}=6.0021\times 10^{4}\;\left(PC\right)$. The relationship between concentration and intensity is given in Figure 11. It was possible to fit the relationship to a linear model $I_{n}=0.00140c+0.5112$ with RMSE=0.005974 and $R^{2}=0.9836$. From the statistical model, it is possible to infer that the biosensor can range up to values of 289 mg/dL.

## 3. Conclusions

Using TEOS to immobilize GOD allows the construction of a glucose biosensor. In general, enzymes have excellent functional properties, in our case the GOD maintains its ability to catalyze even if it is immobilized in the porous matrix. The GOD can be added in the hydrolysis phase without suffering degradation. The biosensor coupled with the PMMA optical fiber exhibits a wide linear range (100–200 mg/dL) and a good sensitivity (0.001403 mg/dL). The range used to evaluate this optical device corresponds to clinical levels of blood sugar, below 100 mg/dL is normal, and 200 mg/dL or higher after two hours suggests diabetes. Thus, this sensor can be used in medical applications. The gelation of TEOS sol–gel, coupling the biosensor to a redox detection using the GOD/HRP configuration, was carried out under the same conditions and proved to be efficient for qualitative visible detection of glucose in solution. The color changes are fully visible to the naked eye, even at concentrations of 2 mM of glucose. Furthermore, the configuration of the detection system allows for the estimation of the enzymatic activity of GOD/HRP based on changes in power and computing the sample’s absorbance over the reaction time. Thus, the qualitative traditional colorimetric method based on an enzymatic reaction can be employed as a quantitative assay for enzymatic activity. The proposed sensor exhibits strong selectivity and stability. Due to its rapid response, the sensor is capable of real-time glucose detection and can be coupled with visual detection. Our proposal to implement an optoelectronic system for estimating enzymatic activity has several advantages. In biochemical engineering, direct measurement devices such as spectrophotometers or fluorometers are used to measure absorbance or absorbance and light emission, respectively; chromatography is also employed to identify and quantify compounds in a sample, thereby allowing the identification of changes in the chemical composition of a substrate. The arrangement shown in Figure 2 proposes an indirect measurement model based on the absorbance of a second product of the enzyme cascade reaction of GOD-HRP. Although further calculations and enzyme concentration assays, such as the Bradford assay, would be required to separate and estimate the individual enzymatic activity of GOD and HRP, our system can estimate the combined enzymatic activity through a simple colorimetric assay. Furthermore, the equipment used, such as the 532 nm green light source and the optical photodetector for measuring light power, result in a much more economical and versatile method compared to the aforementioned devices. By having control over the light source, controlling the light saturation towards the detector, and controlling the power measurement scale, the concentration range of the sample is expanded, allowing for working with high- and low-concentration samples. Unlike traditional equipment, except for fluorometers that measure light emitted by a sample with intrinsic or added fluorescence properties, the use of redox-type dyes provides visual control to monitor different concentrations. In future works, this concept can be built as an integrated optics, avoiding the use of external light sources and complex measurement devices.

## 4. Materials and Methods

The sol–gel process is a synthesis method in which, starting from molecular precursors such as metal alkoxides or inorganic salts, an oxide skeleton is obtained through hydrolysis and polymerization reactions at low temperatures, which allows the synthesis of metastable phases of rust and even mixed organic–inorganic solids [24]. The chemicals used were:

- Tetraethyl orthosilicate (TEOS) 98%; CAS 78-10-4 (Sigma-Aldrich, St. Louis, MO, USA);
- Glucose oxidase (GOD), type X-S from Aspergillus niger, lyophilized powder; CAS 9000137-0 (Sigma-Aldrich, USA);
- Peroxidase from horseradish; CAS 9003-99-0 (Sigma-Aldrich, USA);
- Ethanol CH${}_{3}$CH${}_{2}$OH 98%; CAS 64-17-5 (Sigma-Aldrich, USA);
- Chlorhydric acid HCl 37%; CAS 7647-01-0 (Sigma-Aldrich, USA);
- Acetic sodium salt trihydrate, pH 4.6; CAS 6131-90-4 (Hampton Research, USA);
- Distilled water.

All the chemicals were used without any further purification. The equipment used was:

- Beaker, 20 mL;
- Magnetic stirrer with hot plate;
- Analytical balance, 0.1 mg precision;
- Adjustable micropipette, 0.1 μL–1 mL;
- Hand-held optical power meter, brand Newport, model 1918-C;
- Silicon photodetector, brand Newport, model 918D-SL-OD1R;
- Dip coater.

### 4.1. Sol–Gel Synthesis

The sol–gel process involves the precursor $Si{\left(OC_{2}H_{5}\right)}_{4}$ (TEOS), ethanol, and water. TEOS and water are not miscible; thus, to produce the hydrolysis (Equation (1)) they are mixed in a mutual solvent, in our case, ethanol. After partial hydrolysis, free silanol (Si-OH) groups appear. Eventually, two silanol or one silanol and an ethoxy group condense (Equations (4)–(6)) to form a siloxane (Si-O-Si) group.

Si(OEt)_4_ + H_2_O  ⟶Si(OEt)_3_OH + EtOH  (4)

Si(OEt)_3_OH + SI(OEt)_4_ ⟶(Si(OEt)_3_-O-Si(OEt)_3_ + EtOH (5)

R-Si-OH + HO-Si-R ⟶R-Si-O-Si-R      (6)

Initially, 10 mL TEOS/ethanol solution was stirred magnetically to form a homogeneous solution. This process takes twenty minutes at room temperature (25 °C). The volume ratio of $CH_{3}CH_{2}OH$ to TEOS was $R_{v}=1$. The mixture needs to be acidified by a catalyst; chlorhydric acid diluted in distilled water to $R_{a}=0.1$ M was used. Then, the diluted HCl was added drop by drop until it reached 0.8 mL. The solution remained in agitation for 20 min, an exothermic reaction occured, and the solution turned clear. In addition, to select the initial concentration of chlorhydric acid, a pH test was carried out on the mixture until obtaining a value greater than 5 and less than 7, allowing an adequate acid medium for the enzyme.

Depending on the concentration, the solution will gelate at room temperature from some days to weeks. The addition of a basic solution can speed up this process, but the physical properties of the coating will be dramatically changed. Here, an acid medium was preferred, the gel was dried by evaporation to finally obtain a xerogel.

### 4.2. Immobilization of Enzyme

The most general immobilization procedure is a simple entrapment of the enzyme in a natural or synthetic polymer-forming gel. The main drawback of this technique is the loss of the enzyme by filtration through a non-uniform network of polymer molecules. The rather weak interactions between the enzyme and the matrix result in relatively unrestricted diffusional movement of the polypeptide chains. This can be beneficial when conformational transitions are required for successful catalysis. Otherwise, this freedom of diffusional movement may adversely affect the immobilized enzyme’s stability [25,26]. The datasheet recommends preparing a solution of glucose oxidase (GOD) diluted with a sodium acetate buffer 50 mM, pH 5.1 at 35 °C. However, the sodium acetate will decrease the pH and thus the gelation time. On the encapsulation tests, the enzyme was added (as a powder) directly to the solution. An additional 0.8 mL of HCl (0.1 M) was necessary to regulate the pH between 5 and 7, for 1 mg of GOD the stirring process was maintained for 16 h. To find drying conditions with no detrimental effect on the enzyme, two environmental conditions were tested. The first one left the bioptrodes at room temperature and the second one left them in refrigeration at 8 °C. The aims of this test were to find the parameters to preserve substantial biological activity, and to adjust the mechanical properties of the coating. The experiment revealed that enzymes can be added in the earlier hydrolysis stage or the later condensation stage with no significant loss in enzymatic activity. Nonetheless, at a high volume-percentage of R-OH in the sol–gel solution, the enzyme will be deactivated.

### 4.3. Sol–Gel/GOD Deposition

**Figure 12:**
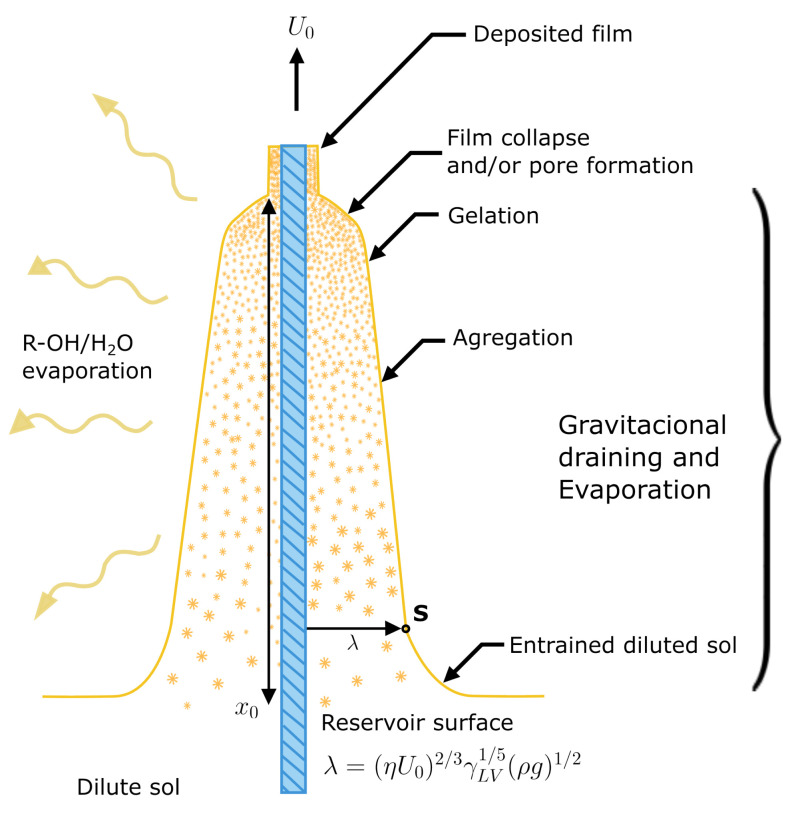
Schematic of the dip-coating process. Adapted from [29].

The optrodes were prepared using cuttings of 2 cm length of plastic optic fiber from Sigmund Optics with a diameter of 2000 um. Finally, 0.5 cm of cladding was removed to expose the fiber core. To strip the fiber [27], chemical etching in acetone was used for 1 min, and then it was gently agitated in soapy water. The coating process used in this work was dip coating [28], a common technique to create thin film coatings. In Figure 12, a schematic representation of the dip-coating process is shown [29]. $U_{0}$ is the withdrawal speed, λ the film thickness in the stagnation point S, η is the solution viscosity, ρ the solution density, $\gamma_{VL}$ is the liquid–vapor surface tension, and g is the gravity.

The process can be separated into the following stages [30]:

- Immersion: The substrate is immersed in the coating material solution at a constant rate (preferably without fluctuations).
- Start-up: The substrate has been sitting in the solution for a while and rises.
- Deposition: The thin layer is deposited on the substrate while being lifted. The withdrawal is carried out at a constant speed. The speed determines the coating thickness, faster removal provides a thicker coating of material [31].
- Drainage: By gravity, excess liquid is drained from the surface.
- Evaporation: The solvent evaporates from the liquid, forming a thin layer. For volatile solvents, such as alcohols, evaporation begins already during the deposition and drainage steps.

To model the thickness evolution during the dip-coating process, many forces in the film deposition must be taken into account. Typical thicknesses for sol–gel coating [32] are around 1–2 μm. After testing different immersion/extraction velocities, the most uniform film results were produced using 1 cm/s for deposition and 0.3 cm/s for drainage. The evaporation velocity was controlled by isolating the optrodes in a refrigerator at 8 °C for 16 h.

### 4.4. Fixation of GOD/HRP to Sol–Gel on Coverslips

The clear sol–gel protocol was carried out. Once the sol–gel was fixed on the coverslip, it was soaked in a solution with the GOD and HRS enzymes containing 50 mM sodium acetate buffer at a pH of 5.1, GOD 1 mg/mL, and HRS 1 mg/mL, and then refrigerated for 30 min. Subsequently, the sol–gel loaded with the enzymes was washed with distilled water and allowed to dry at room temperature.

### 4.5. Colorimetry Samples

The manufacturer’s SIGMA protocol for the GOD activity assay was modified [33,34]. The reaction occurs as shown in Equations (7) and (8).

(7) $$\beta -D-\mathrm{Glucose}+H_{2}O\overset{\mathrm{GOD}}{\to }D-\mathrm{Glucono}-1,5-\mathrm{Lactone}+H_{2}O_{2}$$

(8) $$H_{2}O_{2}+o-\mathrm{Dianisidine}(\mathrm{reduced})\overset{\mathrm{HRP}}{\to }o-\mathrm{Dianisidine}(\mathrm{oxidized})$$

Six sample solutions, containing 10 μM o-Dianisidine in 50 mM sodium acetate buffer at pH 5.1, were prepared in crystalline plastic cuvettes. Once the cuvette was placed in the measurement system, the coverslip with the loaded sol–gel was introduced, and the volume of the solutions was adjusted to 8 mL with sequential glucose concentration gradients of 0, 2, 4, 6, 8, and 10 mM, starting from a 10% glucose solution. The reaction started when the glucose solution was introduced under constant stirring. The samples were measured at a wavelength of 532 nm. 

## Figures and Tables

**Table 1:** Volumetric composition used in the analysis of experiments.

Sample	TEOS	CH${}_{3}$CH${}_{2}$OH	Water	HCl (0.1 M)
R1	5 mL	5 mL	0 mL	0.8 mL
R2	5 mL	5 mL	0.4 mL	0.8 mL
R3	5 mL	5 mL	0.8 mL	0.8 mL
R4	5 mL	5 mL	1.6 mL	0.8 mL

## Data Availability

The data that support the findings of this study are available on request from the corresponding author. The data are not publicly available due to privacy or ethical restrictions.
